# Phase II trial demonstrates the efficacy and safety of individualized, dosimetry-based ^177^Lu-DOTATATE treatment of NET patients

**DOI:** 10.1007/s00259-022-05786-w

**Published:** 2022-04-22

**Authors:** Anna Sundlöv, Katarina Sjögreen Gleisner, Jan Tennvall, Michael Ljungberg, Carl Fredrik Warfvinge, Kajsa Holgersson, Andreas Hallqvist, Peter Bernhardt, Johanna Svensson

**Affiliations:** 1 Department of Clinical Sciences, Oncology and Pathology, Skåne University Hospital, Lund University, Lund, Sweden; 2 Department of Medical Radiation Physics, Clinical Sciences Lund, Lund University, Lund, Sweden; 3 Department of Oncology, Sahlgrenska University Hospital, Blå Stråket 2, 413 45 Gothenburg, Sweden; 4 Department of Oncology, Institute of Clinical Sciences, Sahlgrenska Academy, University of Gothenburg, Gothenburg, Sweden; 5 Department of Radiation Physics, Institute of Clinical Sciences, Sahlgrenska Academy, University of Gothenburg, Gothenburg, Sweden; 6 Department of Therapeutic Radiation Physics, Sahlgrenska University Hospital, Gothenburg, Sweden

**Keywords:** Dosimetry, Radionuclide therapy, ^177^Lu-DOTATATE, Neuroendocrine tumors

## Abstract

**Purpose:**

Radionuclide therapy with ^177^Lu-DOTATATE is well established for patients with advanced somatostatin receptor–positive neuroendocrine tumors with a standard schedule of 7.4 GBq at four occasions. However, this approach does not consider individual variability affecting the tumor radiation dose or dose to organs at risk. Therefore, it is important to assess more personalized strategies. The aim of this phase II trial was to evaluate individualized ^177^Lu-DOTATATE for which the number of cycles varied based on renal dosimetry.

**Methods:**

Patients were eligible if they had a progressive, somatostatin receptor–positive neuroendocrine tumor with a Ki 67 labeling index < 20%. They received cycles of 7.4 GBq of ^177^Lu-DOTATATE at 10 ± 2-week intervals until a predefined radiation dose to the kidneys was reached. The primary endpoint was objective tumor response (RECIST v 1.1). Secondary endpoints included progression-free survival (PFS), overall survival (OS), and toxicity (CTCAE v. 4.0).

**Results:**

Ninety-six patients who had received a median of 5 cycles (range 1–9) were evaluable for efficacy. The objective tumor response was 16% partial response, 66% stable disease, and 19% progressive disease. The median PFS and OS were 29 months and 47 months, respectively, and were significantly associated with kidney dose, performance status, and Ki 67 levels but not with tumor origin. The overall toxicity was mild, and the most common events were grade 1–2 anemia, thrombocytopenia, fatigue, nausea, and diarrhea. Grade 3–4 toxicity occurred in < 10% of patients and was mostly hematological, with no grade 3–4 renal toxicity.

**Conclusion:**

Individualized treatment with ^177^Lu-DOTATATE based on renal dosimetry is clearly feasible with low toxicity and promising efficacy, showing the potential to further improve outcome beyond the standard approach, and should be further assessed in randomized trials.

**Trial registration:**

EudraCT 2011–000,240-16. NCT01456078. https://clinicaltrials.gov/ct2/show/NCT01456078

## Introduction

Peptide receptor radionuclide therapy (PRRT) using ^177^Lu-DOTATATE is a valuable treatment option for patients with somatostatin receptor–positive neuroendocrine tumors (NETs). A somatostatin analog (octreotate) coupled to the radionuclide ^177^Lu binds to somatostatin receptors overexpressed on tumor cells, thus delivering ionizing radiation in a molecularly targeted radiotherapy approach. The treatment effect is due to DNA damage by the beta-particles emitted from ^177^Lu, whereas gamma radiation enables imaging for uptake mapping and dosimetry.

PRRT is endorsed by the major neuroendocrine societies [1–3] and approved for treatment of gastroenteropancreatic tumors (GEPNETs) (by the European Medicines Agency and the US Food and Drug Administration). The evidence of the efficacy and safety of ^177^Lu-DOTATATE was long based on retrospective and single-arm studies [4, 5]. In 2017, this evidence was complemented with the results from the NETTER-1 trial, a randomized phase III trial where the superiority of ^177^Lu-DOTATATE over somatostatin analogs (SSAs) was demonstrated for small intestinal NETs (SiNETs), the largest subgroup of GEPNETs [6].

The approved treatment approach of using a fixed activity of 7.4 GBq ^177^Lu-DOTATATE in four cycles is safe but not necessarily the most effective treatment for individual patients, nor is it in line with current European legislation [7]. In radiotherapy, dose–response relationships are proven for most tumors [8], and the aim is to deliver a sufficiently high dose to the tumor with acceptable exposure to the organs at risk. The same type of dose–response/dose-toxicity relationships can be applied to radionuclide therapy, as is being confirmed by a growing body of evidence through the use of image-based dosimetry in prospective clinical trials [9–14].

The main organs at risk are the kidneys and bone marrow. While bone marrow toxicity presents early and is therefore readily detected, renal toxicity occurs months to years after treatment. The exposure of the kidneys in PRRT is due to an active reabsorption of ^177^Lu-DOTATATE in the renal proximal tubules [15], which can be partially inhibited by a parallel infusion of amino acids [16]. Previous experience with PRRT using ^90^Y has demonstrated dose-dependent renal toxicity at a biologically effective dose (BED) of 28 Gy for patients with risk factors for renal toxicity and 40 Gy for those without risk factors [17]. A renal BED above 45 Gy was correlated with a high risk of rapid decline in renal function [18]. The radiation exposure of the kidneys therefore needs to be monitored. Carrying out as many treatment cycles as possible within a predefined dose limit to the kidneys is a first step towards dosimetry-based, individualized PRRT.

To improve the efficacy of ^177^Lu-DOTATATE, the concept of an individually optimized treatment strategy needs to be further pursued and could be developed by adjusting the injected activity, modifying the treatment intervals, or increasing the total number of cycles. The latter strategy was assessed in the present phase II trial (ILUMINET), where the safety and efficacy of individualized ^177^Lu-DOTATATE treatment based on the estimated renal BED were evaluated. Here we present the final results.

## Material and methods

### Patients

The main eligibility criteria were histologically verified irresectable neuroendocrine tumors irrespective of origin, with a Ki 67 labeling index of ≤ 20% and an ECOG performance status (PS) of 0, 1, or 2. The disease had to be progressive on CT scan during the last 14 months. Tumor uptake higher than basal liver uptake on a ^111^In-Octreotide scan was required. Other inclusion criteria were measurable disease according to RECIST v 1.1 criteria, adequate bone marrow and liver function, a measured glomerular filtration rate (GFR) of > 50 ml/min, and a stable dose of somatostatin analog (SSA) during the last 3 months prior to inclusion. Key exclusion criteria were chemotherapy or local treatment during the last 3 months, concomitant nephrotoxic drugs, and previous external beam radiotherapy to > 25% of the bone marrow.

Patients entering step 2 (see “Study design and treatment”) had to have maintained a GFR > 50 ml/min with a maximum decrease of 40% from baseline, no grade 3–4 toxicity, and a maximum age of 70 years. Furthermore, patients were excluded from step 2 if they had a history of diabetes or uncontrolled hypertension or if they previously had received liver embolization or chemotherapy.

The trial was conducted in accordance with ICH GCP and the Declaration of Helsinki and was approved by the regional ethics review board (EPN Lund 2011/287).

### Study design and treatment

The ILUMINET trial was a single-arm, phase II trial conducted at two tertiary referral centers in Sweden. The safety and efficacy of individualized ^177^Lu-DOTATATE treatment based on renal dosimetry were evaluated with the hypothesis that treatment may be optimized by adjusting the number of cycles to the individually estimated renal BED [17, 19]. All patients were planned for treatment up to a cumulative renal BED of 27 ± 2 Gy (step 1). Thereafter, patients complying with the inclusion and exclusion criteria for step 2 (see “Patients”) were offered further treatment up to a renal BED of 40 ± 2 Gy.

Treatment was administered as intravenous infusions of 7.4 GBq ^177^Lu-DOTATATE, at 10 ± 2-week intervals, preceded by antiemetics and co-administered with a kidney-protective amino acid infusion (2 L VAMIN® 14 g N/l starting 30 min before treatment and continued for 8 h). Long-acting SSA was withheld at least 4 weeks before the administration of each cycle. For dosimetry, four planar whole-body scintigraphies (1 h, 24 h, 48 or 96 h, and 168 h post infusion) and one combined SPECT/CT (24 h) were performed, from which the BED was calculated as previously described [20].

### Endpoints and assessments

The primary endpoint was objective tumor response 3 months after completing step 1, based on RECIST v 1.1 criteria. The main secondary endpoints were progression-free survival (PFS), overall survival (OS), toxicity according to CTCAE v. 4.0 criteria, and health-related quality of life. Renal toxicity was considered an adverse event (AE) of special interest and is therefore explained separately. Exploratory endpoints included the effect of renal BED, Ki 67, PS, and tumor origin on PFS and OS, as well as best overall response, time to maximum response, and time to progression (TTP). All time-to-event endpoints were estimated from date of inclusion. For comparison of efficacy based on renal BED, the delivered (rather than the targeted) BED was used to group the patients into “ < 25 Gy,” “25–29 Gy,” and “ > 29 Gy.”

Patients were followed during the treatment phase and follow-up with CT scans of the thorax and abdomen every 3 months, including RECIST evaluation. Plasma creatinine and an estimated GFR were determined at each follow-up visit, and a GFR measurement was performed yearly. Peripheral blood values were assessed weekly for 6 weeks after each treatment cycle, before each new cycle, and at 3-month intervals during follow-up. Toxicity was analyzed separately for early (from the start of therapy to 3 months after completing step 1) and late (6 and 12 months after the last treatment) adverse events.

Bone marrow (BM) dosimetry was also conducted with a previously described image-based methodology, and health-related quality of life was assessed with the EORTC QLQ-C30 and EORTC QLQ-GI NET21 questionnaires. These data will be analyzed and reported separately.

### Statistical analysis

Based on an expected objective response rate of 20–40%, 100 patients were needed to obtain a 95% confidence interval with a margin of error of 10%.

Descriptive statistics are presented using the median and range for continuous variables and the count and percentage for categorical variables. Univariate analysis was performed on PFS and OS dependence on stratification on kidney BED, ECOG and Ki 67.

Confidence intervals for proportions were calculated using the Wald method. Kaplan–Meier curves were used to evaluate OS and PFS. Log rank tests were used to test for differences in OS and PFS between subgroups. All statistical analyses were performed in R version 4.0.2 [21].

## Results

### Patients and treatment

Between October 2011 and June 2018, 97 patients were enrolled. One patient never received treatment due to withdrawal of consent. The baseline characteristics of the remaining 96 patients are shown in Table 1.

**Table 1:** Demographics and baseline characteristics

Variable	Step 1 (*n* = 96)	Step 1 + 2^a^ (*n* = 9)
Age, median [min–max]	67 [35, 84]	59 [44, 69]
Sex, count (%)		
Male	54 (56)	5 (56)
Female	42 (44)	4 (44)
Ki67, count (%)		
0–2	36 (38)	3 (33)
3–10	43 (45)	5 (56)
11–20	17 (18)	1 (11)
Tumor origin, count (%)		
Small intestine	57 (59)	3 (33)
Pancreas	18 (19)	1 (11)
Other^b^	21 (22)	5 (56)
ECOG, count (%)		
0	55 (57)	6 (67)
1	30 (31)	2 (22)
2	5 (5)	-
Missing	6 (6)	1 (11)
Time from diagnosis (months), median [min–max]	48 [1.9, 207]	50 [1.9, 207]
Previous treatment, n(%)	Step 1	Step 1 + 2
None (except SSA)	9 (9)	2 (22)
Chemotherapy	16 (17)	0
Sunitinib/everolimus/interferon	18 (19)	0
Surgery	75 (78)	7 (77)
PRRT	0	0
Liver directed	44 (46)	0
Laboratory findings, median [min–max]	Step 1	Step 1 + 2
Creatinine	77 [47–146]	73 [58–102]
GFR	75 [48–122]	85 [72–96]
Hb	135 [97–165]	140 [122–165]
LPK	6.0 [2.4–20]	5.4 [3.7–8.0]
ANC	3.5 [1.6–11]	3.3 [1.9–4.7]
TPK	222 [52–552]	209 [157–344]

^a^9 out of the 96 patients treated according to step 1, who proceeded to step 2

^b^Other tumor origins included the lung (*n* = 9), unknown primary tumor (*n* = 5), colon (*n* = 3), rectum (*n* = 2), and stomach (*n* = 2)

Because there were five deaths before radiologic assessment and one case of protocol deviation, 90 patients were evaluable for the primary endpoint of objective tumor response. The total population of 96 patients was evaluable for OS and toxicity, and 94 patients were evaluated for PFS and TTP. Ninety patients were evaluable for the exploratory objectives of time to maximum response and best overall tumor response.

Thirty-two patients terminated treatment before completing step 1. The reasons for termination were death (*n* = 7), toxicity (*n* = 13), and progressive disease (*n* = 12). Of the 64 patients completing step 1, nine continued to receive additional treatment in step 2. The median number of treatment cycles for all patients was 5 (1–9), with 51 patients (53%) receiving more than four cycles. For comparison of efficacy, patients were grouped based on received renal BED: < 25 Gy (*n* = 61), 25–29 Gy (*n* = 24), and > 29 Gy (*n* = 11).

### Efficacy

**Fig. 1:**
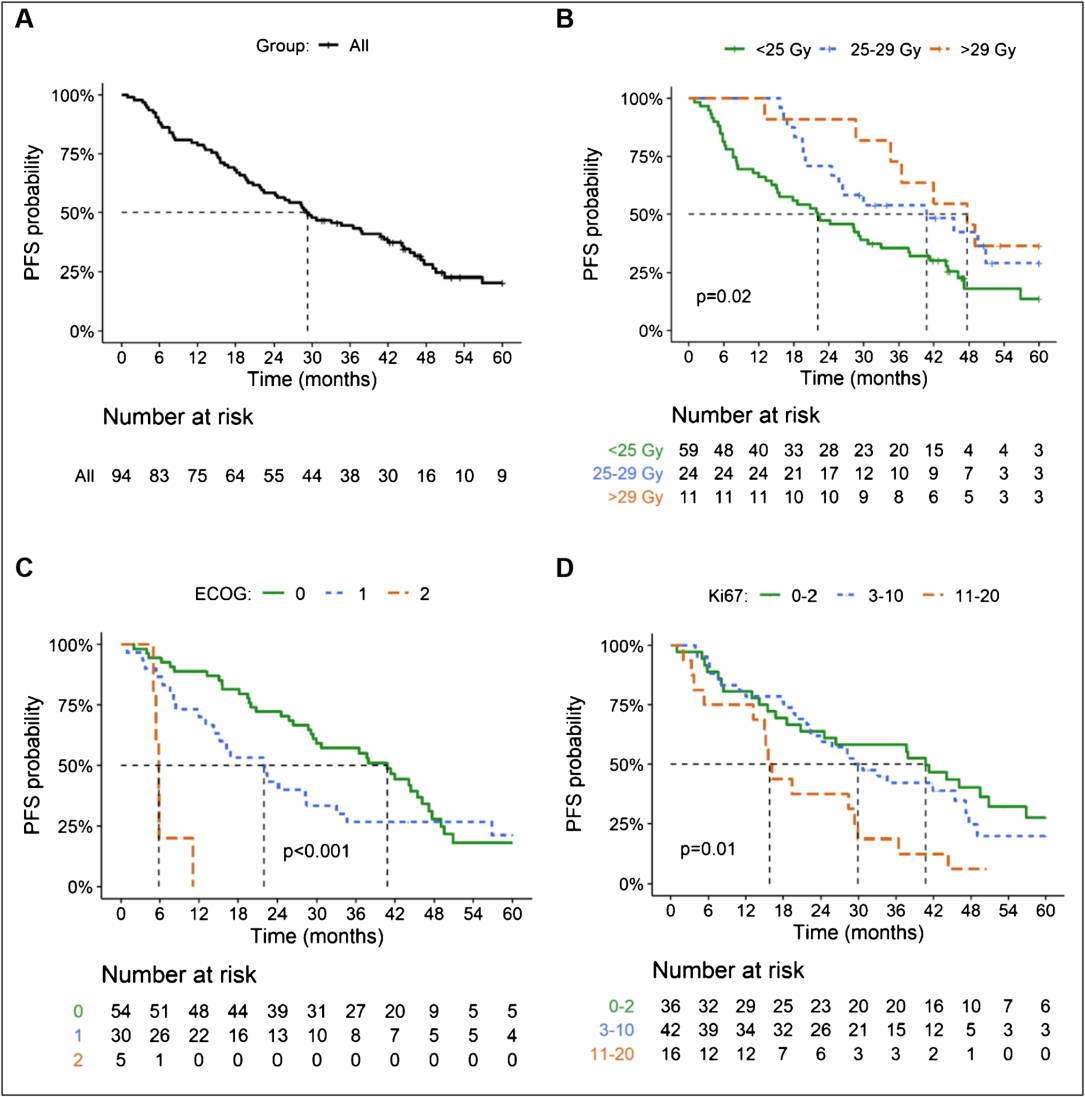
Kaplan–Meier plots of progression-free survival for all (**a**), by BED (**b**), baseline ECOG (**c**), and Ki 67 labeling index (**d**)

**Fig. 2:**
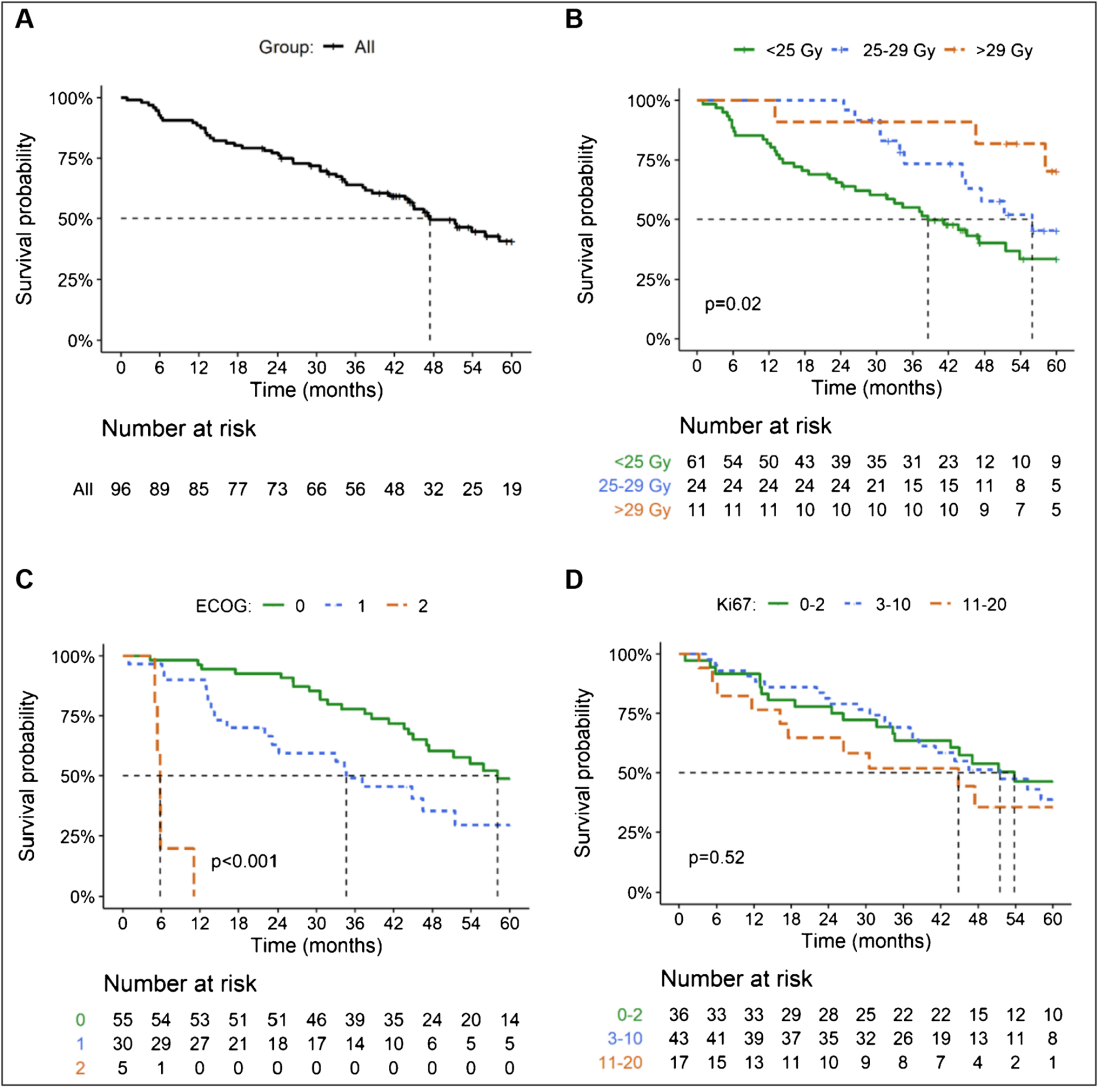
Kaplan–Meier plots of overall survival for all patients (**a**), by BED (**b**), baseline ECOG (**c**), and Ki 67 labeling index (**d**)

**Fig. 3:**
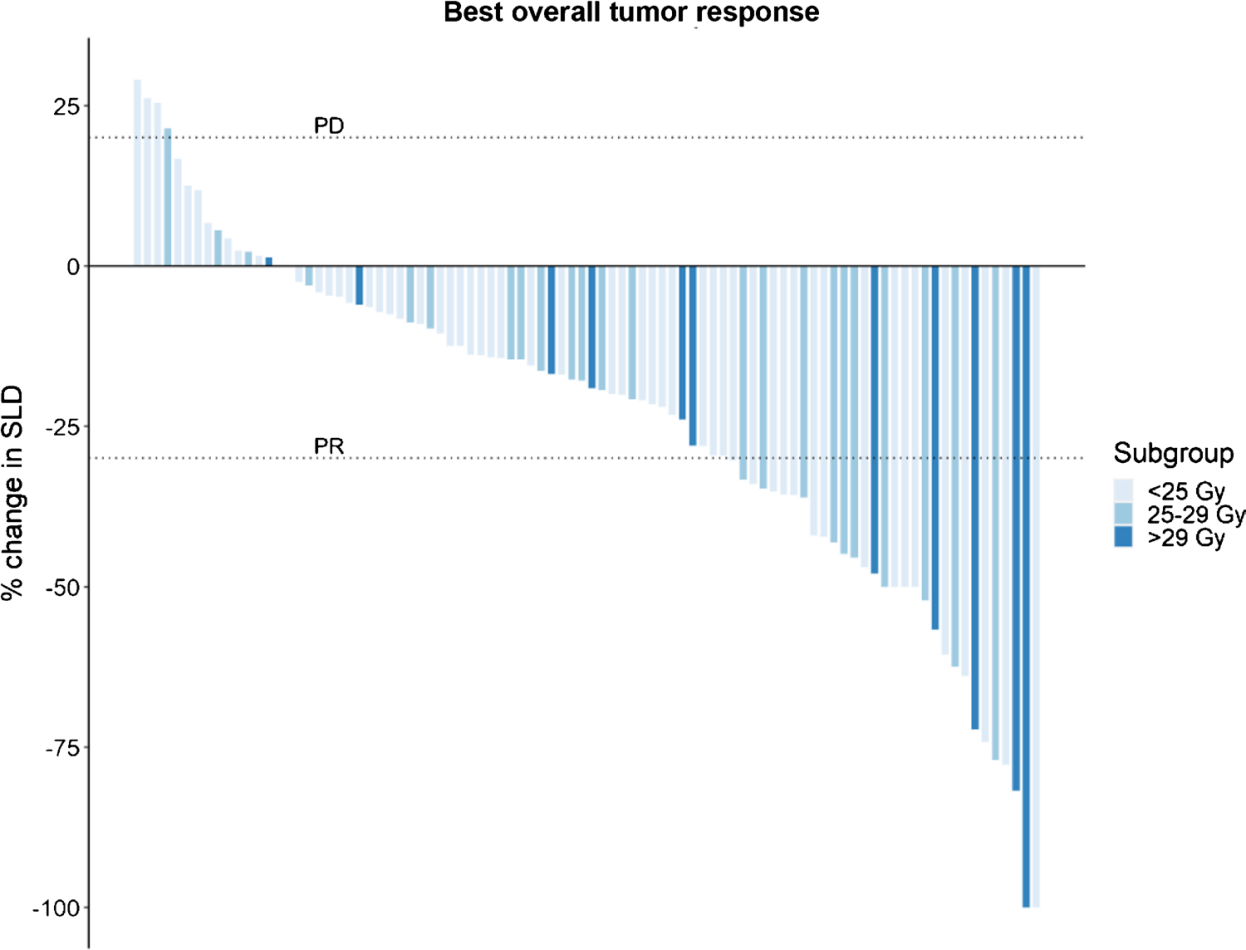
Percentage change in sum of longest diameters (SLD) in target lesions from baseline to post-baseline nadir grouped by renal BED

The median follow-up at the time of analysis was 42 months. The objective tumor response 3 months after step 1 was 16% partial response (PR) (95% CI, 8.1 to 23%), 66% stable disease (SD) (95% CI, 56 to 75%), and 19% progressive disease (PD) (95% CI, 11 to 27%). The PFS and OS were 29 months and 47 months, respectively (Figs. 1a and 2a), with a 5-year survival of 41% (95% CI, 31 to 54%). When analyzing PFS and OS, there were significant differences according to renal BED and ECOG (Figs. 1b, c and 2b, c), while for Ki67, there were differences for PFS but not for OS (Figs. 1d and 2d). There were no significant differences in median PFS or OS according to tumor origin (data not shown). The best overall response (including step 2) was 2% complete response (CR) (95% CI, none to 5%), 32% PR (95% CI, 23 to 42%), 61% SD (95% CI, 51 to 71%), and 4% PD (none to 9%) (Fig. 3).

The median time to maximum response was 18 months for all patients (95% CI, 16 to 22) and 13 (95% CI, 11 to 19 months), 23 (95% CI, 18 to 31 months), and 28 months (95% CI, 17 to 36 months) for the three renal BED groups < 25 Gy, 25–29 Gy, and > 29 Gy, respectively. The median TTP was 41 months (95% CI, 29 to 48 months) for all patients and 31 (95% CI, 22 to 46 months), 46 (95% CI, 26 months to not reached), and 48 months (95% CI, 36 months to not reached) for the renal BED groups < 25 Gy, 25–29 Gy, and > 29 Gy, respectively. The differences in TTP based on stratification were not significant.

### Safety

The overall toxicity was mild, with few grade 3–4 adverse events (AEs). AEs are summarized in Table 2.

**Table 2:** Adverse events

Early adverse events			Late adverse events		
Clinical AE	All grades, *n* (%)	Grade 3–4, *n* (%)	Clinical AE	All grades, *n* (%)	Grade 3–4, *n* (%)
Fatigue	60 (62)	1 (1.0)	Fatigue	9 (9.4)	-
Nausea/Vomiting	48 (50)	1 (1.0)	Thromboembolic disease	1 (1.0)	1 (1.0)
Pain	21 (22)	1 (1.0)	-	-	-
Diarrhea	19 (20)	-	-	-	
Abdominal pain	18 (19)	1 (1.0)	-	-	-
Flushing	7 (7.3)	-	-	-	-
Alopecia	6 (6.2)	-	-	-	-
Constipation	5 (5.2)	-	-	-	-
Depression	5 (5.2)	-	-	-	-
Infection	5 (5.2)	1 (1.0)	-	-	-
Ileus	2 (2.1)	1 (1.0)	-	-	-
Thromboembolic disease	2 (2.1)	2 (2.1)	-	-	-
Weight loss	2 (2.1)	1 (1.0)	-	-	-
Biliary tract infection	1 (1.0)	1 (1.0)			
Dehydration	1 (1.0)	1 (1.0)			
Laboratory AE	All grades (%)	Grade 3–4, ***n*** (%)	Laboratory AE	All grades, n (%)	Grade 3–4, n (%)
Thrombocytopenia	58 (60)	9 (9.4)	Anemia	20 (21)	-
Anemia	51 (53)	1 (1.0)	Thrombocytopenia	10 (10)	-
Leucopenia	32 (33)	4 (4.2)	Neutropenia	9 (9.4)	1 (1.0)
Neutropenia	28 (29)	6 (6.2)	Leukopenia	8 (8.3)	1 (1.0)
Liver enzyme increase	6 (6.2)	1 (1.0)		-	-

*AE* adverse event. Early AEs included events from the start of therapy to 3 months after completing step 1 and late AE events 12 months after the last treatment

In terms of early AE, the most common (> 5%) clinical AEs were grade 1–2 fatigue, nausea, pain, diarrhea, abdominal pain, flushing, and alopecia. Grade 3 events were reported in single patients experiencing thromboembolic disease, nausea, and pain. No grade 4 clinical AEs were registered. Hematological AEs were common, with grade 1–2 anemia and thrombocytopenia occurring in more than half of the patients. Grade 3–4 laboratory findings were observed in 1–9% of patients, including hematological AEs and liver enzyme increase.

Regarding late AEs, the only clinical AE occurring in > 5% of the patients and persisting 12 months after treatment was grade 1 fatigue. There was one grade 3–4 clinical AE, a thromboembolic event. The only persisting laboratory findings were grade 1–2 hematological AEs, whereas all grade 3–4 AEs occurred in < 5% of the patients at 12 months. During follow-up, two patients were diagnosed with acute myeloid leukemia 2 and 4 years after the first cycle of ^177^Lu-DOTATATE. The patients were 69 and 76 years old, had received no systemic treatment other than SSA against their SiNETs, and were treated with 4 and 6 cycles, respectively.

Among the seven patients who completed treatment in step 2, there was no grade 3–4 AE, neither early nor late clinical or hematological/biochemical. All seven patients had a GFR > 60 ml/min at baseline, and four of them maintained this level at the time of last follow-up. The remaining three patients had a last measured GFR of 30–60 ml/min. Median follow-up for this subgroup was 45 months.

Twelve patients terminated the study before completing step 1 due to AEs, 11 due to hematological toxicity (10 had grade 1–3 thrombocytopenia together with either grade 2–3 neutropenia or grade 1–3 anemia; one patient had grade 3 neutropenia as the only reason for early termination of treatment). Three of the 11 patients had received 5 cycles of treatment, and the remaining eight had received only 2–3 cycles, with three of these having been pre-treated with radiation- or chemotherapy. The patients that terminated treatment early did not have a higher median age nor had they received more lines of treatment before entering the trial. The presence of bone metastases may have influenced the tolerability due to higher mean absorbed doses, as shown earlier [22], but this remains to be confirmed in the present patient material.

Measured GFR values after terminating the treatment phase were available for 71 patients, with a median follow-up of 29 months. At this point, 59% of patients had a GFR > 60 ml/min (grade 0–1) compared to 82% at baseline, and 39% of patients had a GFR of 30–60 ml/min (grade 2) compared to 18% at baseline. There was one case of grade 3 toxicity related to intercurrent nephrolithiasis. Plasma creatinine levels increased over time, leading to an increase in the frequency of grade 1 toxicity (i.e., p-creatinine 100–150 µmol/L) during follow-up: 17% at baseline vs. 47% at 36 months of follow-up. There was one case of grade 2 renal toxicity and no grade 3–4 events.

## Discussion

The results of this trial show that dosimetry-based PRRT for NET patients is safe and effective. Direct comparisons to the standard treatment used in the NETTER-1 trial are difficult since there are significant differences in the patient populations and trial designs, e.g., the proportion of low-grade NETs (2/3 in NETTER-1 and 1/3 in ILUMINET) and the time to progression prior to trial entry (36 months in NETTER-1 and 14 months in ILUMINET). Despite the higher risk population in ILUMINET, the best overall response rate of 34% compares favorably and the PFS and OS of 29 and 47 months repectively is simular to the results of NETTER-1 (18%, 28 and 48 months, respectively) [23].

Dosimetry-based PRRT has been conducted and reported by two other groups. The Uppsala observational study [24] had a design similar to ILUMINET, but the patient population included high-grade tumors. The number of cycles they administered was 1–10, median not reported, with 48% receiving more than four cycles. They reported a PFS of 27 months, with a response rate of 24%.

A Canadian trial used the same renal dose limit as the Uppsala study (23 Gy), but instead of varying the number of treatment cycles, they adjusted the injected activity in such a way that the renal dose limit was reached in four cycles [25]. This group reported a high response rate of 59% but a surprisingly short PFS of 16 months, and a high rate of, mainly hematological, grade 3–4 toxicity. This raises the question of the importance of the fractionation schedule—is it better to give smaller, repeated doses over a longer period of time than to give the same amount of activity over a shorter period? In the current study, the total median injected activity/patient was 37 GBq, compared to 36 GBq in the study by Del Prete et al. The duration of treatment was up to 97 weeks (median 44) in the current study, and up to 30 weeks in the latter. So, even though the protocol-specified dose constraint to the kidneys differed between the two trials, the total injected activity/patient was similar. There was, however, a large difference in the duration of the treatment period which could theoretically be a reason for the large difference in PFS. In our own material, there is a strong correlation (*R*^2^ = 0.9, *p* < 0.01, data not shown) between cumulative BED and duration of treatment period, so we are not able to differentiate between these two potential explanatory factors for PFS.

The significant differences in PFS when subgrouped by Ki 67, ECOG, and renal BED are challenging to interpret since there are obvious confounders (such as treatment-related toxicity, prognosis, and selection bias). The fact that there was very limited toxicity in the highest BED group, and the poorer results for the patients with the highest Ki 67, would support a strategy in future trials to intensify treatment for the higher grade tumors with the hope of improving outcomes. No significant differences in PFS or OS were seen for different origins; however, pNET patients had a higher Ki 67% than SiNET patients (Table 3). This result has also been reported by others [4] and may reflect that pNETs are more responsive to radiation therapy in general than the more indolent group of SiNETs.

**Table 3:** Baseline and treatment response characteristics grouped by tumor origin

		Primary tumor origin			
		Small intestine (*n* = 57)	Pancreas (*n* = 18)	Other^a^ (*n* = 21)	All (*n* = 96)
Baseline characteristic					
Age, median [min–max]		69 [36–84]	54 [46–76]	63 [35–78]	67 [35–84]
Ki67, median [min–max]		2 [1-20]	10 [1-19]	5 [1-20]	4 [1-20]
ECOG, count (%)	0	31 (54)	9 (50)	15 (71)	55 (57)
	1	19 (33)	7 (39)	4 (19)	30 (31)
	2	4 (7)	0	1 (5)	5 (5)
	Missing	3 (5)	2 (11)	1 (5)	6 (6)
Previous lines of treatment					
SSA only		5 (9)	0	4 (20)	9 (9)
Chemotherapy		0	11 (61)	5 (24)	16 (17)
TKI/ mTORi/INF		7 (12)	8 (44)	3 (14)	18 (19)
Surgery		51 (90)	11 (61)	13 (62)	75 (78)
PRRT		0	0	0	0
Liver-directed		32 (56)	8 (44)	4 (19)	44 (45)
Information collected after baseline					
Subgroup, count (%)	< 25 Gy	39 (68)	12 (67)	10 (48)	61 (64)
	25–29 Gy	14 (25)	5 (28)	5 (24)	24 (25)
	> 29 Gy	4 (7)	1 (6)	6 (29)	11 (12)
Time to max tumor response, median [min–max]		(*n* = 54)	(*n* = 17)	(*n* = 19)	(*n* = 90)
		17 [2–63]	18 [1–48]	22 [3–40]	18 [1.4–63]
Total number of treatments cycles, median [min–max]		4 [1-8]	5 [1-8]	5 [1-9]	5 [1-19]

*TKI* tyrosine kinas inhibitor, *mTORi* mTOR inhibitor, *IFN* interferon. ^a^Other tumor origins included the lung (*n* = 9), unknown primary tumor (*n* = 5), colon (*n* = 3), rectum (*n* = 2), and stomach (*n* = 2).

The results of the analysis of PFS and OS by renal BED beg the question of whether more cycles of treatment result in a higher dose to the tumor and thereby a better and more durable response. This question cannot be definitively answered in a nonrandomized setting, but the fact that the time to maximum response also increased by BED level from 12 months (BED < 25 Gy) to 23 and 28 months (BED 25–29 and > 29 Gy, respectively) implies that with increasing number of cycles the tumor shrinkage continues for a longer time.

When designing the trial, we expected a larger proportion of patients to be able to continue treatment in step 2. Sixty patients (62%) were excluded from step 2 because of earlier chemotherapy or liver-directed treatment (Table 1), and 7 patients (7%) were excluded solely based on age > 70 years. Given the limited toxicity noted in this trial, it seems safe to offer treatment beyond the proposed renal dose limits with less restrictive criteria, with the goal of a better long-term effect and survival.

When designing the trial, it had to be decided which renal dose limits to use based on existing evidence. The commonly used renal absorbed dose limit of 23 Gy originates from external beam radiotherapy (EBRT) and is based on delivering 2 Gy fractions at a dose rate of approximately 1 Gy/min, which has a very different biological effect than the dose rate achieved with PRRT. Assuming that the linear-quadratic model is valid for both EBRT and PRRT, 23 Gy given in 2 Gy fractions corresponds to approximately 40 Gy BED, while an absorbed dose of 23 Gy at the typical dose rate of PRRT corresponds to approximately 25 Gy BED (4.5 Gy/cycle, $\alpha /\beta =2.6$, and repair half-life of 2.8 h assumed) [20]. Furthermore, the renal BED thresholds of 27 and 40 Gy used in the current trial may have been overly conservative, since ^90^Y-PRRT has a more uniform energy deposition pattern than ^177^Lu [26], possibly explaining the more pronounced renal toxicity. Renal function did seem to decline slightly during follow-up, however, which will be further studied in an ad hoc analysis with the goal of determining the relevant renal dose limit for ^177^Lu-PRRT. An important step towards making dosimetry-based treatment generally available is to simplify the imaging procedure on which the dosimetry is based. Single or dual time-point SPECT imaging has been explored by our group and others and found to give reliable dosimetric estimates for the kidneys, especially when the last imaging time-point is at least 72 h after therapy [27–31].

The biological effects of the absorbed radiation dose on tumor lesions and normal tissue in PRRT are likely influenced by several factors governed both by patient (size, renal function, risk factors for toxicity, etc.) and tumor (volume, proliferation rate, intrinsic radiation sensitivity) characteristics. To achieve a truly personalized PRRT, we need to understand which of them have the greatest impact on treatment outcomes and design the treatment accordingly [32]. Regardless of strategy, a personalized approach motivates dosimetry evaluations of organs at risk and ideally of the tumor as well. There are several situations when this information would facilitate treatment decisions, e.g., retreatment with ^177^Lu-DOTATATE, treatment of patients with reduced kidney function or bone marrow reserve, and intensified treatment to more rapidly progressing tumors, e.g., NET G3 with a Ki 67 labeling index > 20%.

For many patients, the standard regimen of four cycles of ^177^Lu-DOTATATE will provide valuable responses and survival benefits. The current results confirm that by individualizing the therapeutic approach the response rate can increase considerably, without causing clinically significant acute or late toxicity. This raises the question whether it is still justifiable to treat according to the currently approved, non-individualized regime. It is possible that it leads to a significant proportion of patients being undertreated, exposing them to the much greater threat to their survival than low-grade toxicity, namely tumor progression. Further improvement of the individualized treatment approach may be achieved by offering patients with more aggressive tumors higher intensity treatment, including combinations of PRRT with other systemic anti-cancer therapies, while patients with low-grade tumors continue to receive a therapy with minimal toxicity and impact on their everyday life. Thus, there is currently a high unmet need for well-designed randomized trials in PRRT, through which the risks and benefits of personalized vs. standard therapy can be demonstrated and quantified.

## Data Availability

The datasets generated and/or analyzed during the current study are available from the corresponding author on reasonable request.
